# Designer Exosomes: Smart Nano-Communication Tools for Translational Medicine

**DOI:** 10.3390/bioengineering8110158

**Published:** 2021-10-26

**Authors:** Madhyastha Harishkumar, Madhyastha Radha, Nakajima Yuichi, Gothandam Kodiveri Muthukalianan, Ohe Kaoru, Koichiro Shiomori, Kentaro Sakai, Watanabe Nozomi

**Affiliations:** 1 Department of Cardiovascular Physiology, Faculty of Medicine, University of Miyazaki, Miyazaki 8891692, Japan; radharao@med.miyazaki-u.ac.jp (M.R.); yunakaji@med.miyazaki-u.ac.jp (N.Y.); 2 School of Biotechnology and Biosciences, VIT University, Vellore 632014, Tamil Nadu, India; k.m.gothandamm@vit.ac.in; 3 Department of Applied Chemistry, Faculty of Engineering, University of Miyazaki, Miyazaki 8892192, Japan; okaoru@cc.miyazaki-u.ac.jp (O.K.); shiomori@cc.miyazaki-u.ac.jp (K.S.); 4 Center for Collaborative Research, Frontier Science Division, University of Miyazaki, Miyazaki 8892192, Japan; k-sakai@cc.miyazaki-u.ac.jp

**Keywords:** exosome, microvesicles, cell engineering, regenerative disease

## Abstract

Exosomes are the master transporters of genes, RNAs, microRNAs, proteins, and lipids. They have applications in major diseases, including cancer, cardiovascular diseases, neurological disorders, and diabetes mellitus. Delivery of the exosomes to recipient cells is governed by the functional heterogenicity of the tissues. Engineered exosomes are promising tools in tissue regeneration. In addition to their role as intracellular communication cargos, exosomes are increasingly primed as standard biomarkers in the progression of diseases, thereby solving the diagnostic dilemma. Futuristic empowerment of exosomes with OMICS strategy can undoubtedly be a bio-tool in translational medicine. This review discusses the advent transformation of exosomes in regenerative medicine and limitations that are caveats to broader applications in clinical use.

## 1. Introduction

In multicellular organisms, a balance between cellular function and tissue homeostasis is mediated by effective intracellular communications by either direct cell to cell interaction or molecular transporter cargos, such as extracellular vehicles (EVs). These master vesicles are derived from endosomes and plasma membranes [1]. Small EVs, also known as exosomes, are released from the cells into the body fluids, which include blood, semen, saliva, urine, breast milk, and cerebrospinal fluids [2,3]. The effective delivery of exosomal components, such as proteins, lipids, RNAs, and DNAs, depends on the interactive ability of the exosomes with the recipient cells. Exosomes are conventionally isolated by differential and buoyant density centrifugation, ultrafiltration, size exclusion chromatography, ligand concentrate method, and immunoaffinity chromatography [4]. Recently, techniques such as polymer-based trapping, micro-fluidics separation, and one-step exo-cap kits have been standardized [5,6]. The exosomes’ size, shape, charge, density, and porosity are prime parameters of utmost importance for understanding the action mode. These parameters are evaluated by various bio-physical techniques, including transmission electron microscopy (TEM), scanning electron microscopy (SEM), atomic force microscopy (AFM), nanoparticle tracking analysis (NTA), dynamic light scattering (DLS), resistive pulse sensing (RPS), enzyme-linked immunosorbent assay (ELISA), flow cytometry, fluorescence-activated cell sorting (FACS), microfluidics, and electrochemical biosensors captures [7].

Secreted exosomes are generally 20 to 200 nm diameter in size and have the similar topology as the parent cell. Specific signals transmitted by the latter governs trafficking of the exosomes to recipient cells; host cell permissiveness, therefore, has novel applications in human health and disease [8]. Most cell types, such as fibroblasts, immune cells, epithelial cells, and endothelial cells secrete exosomes into body fluids. The exosomes are in constant circulation, thereby influencing the physiological and pathological process [9]. Controlling the infections depends on the immune system’s ability to recognize and respond to specific organisms during the pathogenic invasion. Research studies have demonstrated the role of exosomes in promoting or inhibiting host immunity in response to parasitic, fungal, and bacterial infections. Interestingly, exosomes can be exploited by pathogenic prions and viruses to evade the immune response, and hence are implicated in viral particles spreading in the human body, including SARS-COVID-19 [10,11,12]. Pathogen-associated molecular patterns (PAMPs), such as carbohydrates, genetic materials, and proteins from the pathogens are delivered through exosomes to the pattern-recognition receptors (PRRs) and Toll-like receptors (TLRs), found on the target cell surface, thereby initiating the cell-signaling cascade [13]. Functional validation of exosomes depends on the types of cells and loading patterns of metabolites. Table 1 provides the details of functions of exosomes with respect to their origin.

Understanding the positive and negative effects of exosomes is vital in the context of tissue hemostasis. Although the relevance of exosomes in pathophysiology has been studied for the past two decades, insights on molecular loading into exosomes, trafficking of exosomes, and the application of engineered exosomes as tools for targeted drug delivery still requires more attention. This review focuses on the role of exosomes in the pathophysiological arena and the benefits of engineering them to be used as theranostic tools for tissue regeneration.

### Prelims of Exosome Biogenesis and Cargo Loading Patterns

**Figure 1:**
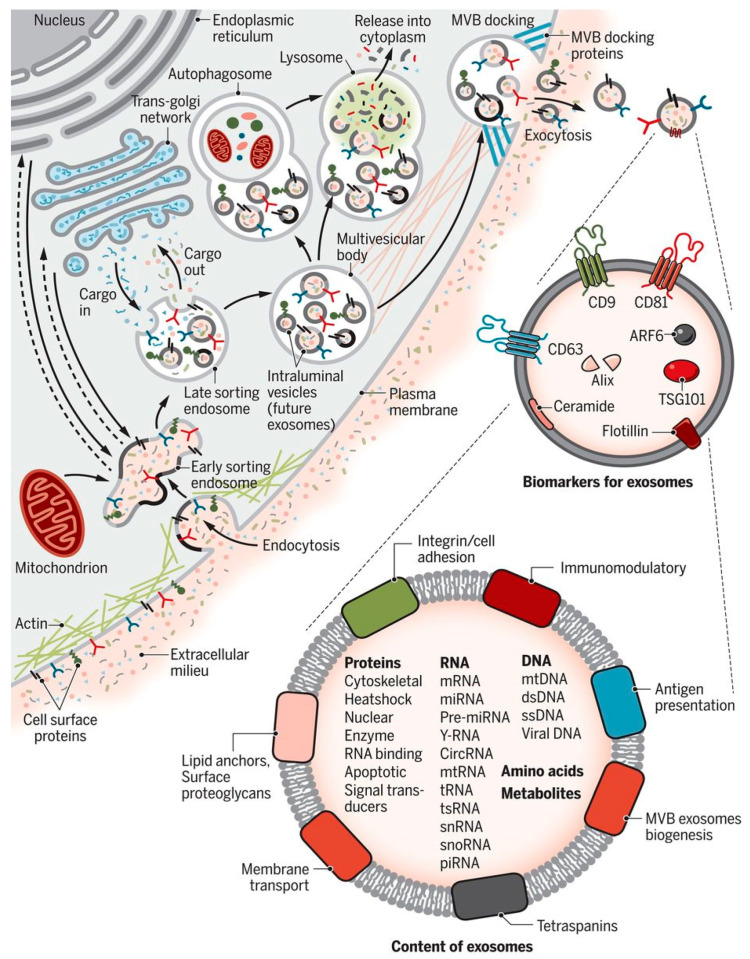
Biogenesis and loading pattern of exosomes. The key steps of exosome biogenesis include the endocytosis of metabolite endocytosis into the cell, formation of exosomes in the lumen of plasma membrane, and exocytosis of the loaded exosomes. Figure is taken from [35], copyright from the American Association of Advancement of Sciences.

Numerous hypotheses are proposed for the formation, initiation, and maturation of exosomes. Biogenesis starts with the interaction between syntenin and syndecan proteins (SDCs) with proteins such as ALG-2 interacting protein X (ALIX) and CHMP4 interacting with endosomal sorting machinery, namely the endosomal sorting complexes required for transport (ESCRT) [39,40], to initiate the process of genesis. ALIX and ESCRT work synergistically towards protein sequestration, modification, processing, trafficking to the respective vesicles, and subsequent fusion with the plasma membrane [16]. Syndecans (SDCs) are type-1 integral membrane heparan sulphate proteoglycans (HSPGs) composed of four different genes (SDC 1-4). The master gene SDC 4 regulates several vesicular trafficking molecular pathways together with syntenin and the adaptor protein Bro1/ALIX [32]. These mechanisms of biogenesis have been the subject of vigorous debate in recent years. However, it is now clear that the first step of membrane invagination requires ALIX, syntenin, and syndecan and that the SDC-4 gene is actively involved in intraluminal vesicle formation and selection of cargo in an ESCRT dependent mechanism. ESCRT independent mechanisms involving lipids and tetraspanins also assist exosome formation [41]. Together with transmembrane and cytosolic proteins, these lipoprotein superfamilies mediate the organization of tetraspanins-enriched microdomains (TEMS) in the plasma membrane and help biogenesis of exosomes, as described by Hemler [42]. Ceramide, a simple spingo-lipid (SL) molecule, plays an essential role in the ESCRT independent exosome biogenesis mechanism. In the endoplasmic reticulum (ER), L-serine and palmitoyl-CoA are condensed by serine palmitoyl-transferases (SPT) to form long SL bases. The bases are reduced by 3-ketospinganine reductase and N-acylation of ceramide by dihydroceramide desaturases form the exosome blubbing [43]. The details of the biogenesis and exosome loading patterns are depicted in Figure 1 [35]. Extra cellular components such as proteins, lipid moieties, and metabolites enter the cells by endocytosis through the cell membrane. Bud formation takes place in the luminal side of the membrane, and this bud eventually forms into micro vesicles as first step of biogenesis. The key proteins involved in exosome biogenesis include Rab GTPases, ESCRT proteins, and others that are also used as markers for exosomes (CD9, CD81, CD63, flotillin, TSG101, ceramide, and ALIX). Exosome surface proteins include tetraspanins, integrins, and immunomodulatory proteins. Exosomes can contain different cell surface proteins, intracellular proteins, RNA, DNA, amino acids, and metabolites, and the content can vary with different bio-metabolites [15,35].

## 2. Scenario Oof Exosomes in Translational Medicine

### 2.1. Relationship between Cancer and Exosomes

The rate of exosome production and their release from cancer cells exceeds that of the normal cells. First reported in 1983, the research on exosomes in cancer biology and metastasis has expanded exponentially [44]. Exosomal cargo (RNA, miRNA, proteins, DNA, and small apoptotic bodies) significantly changes recipient cells’ fate and genetic signature. Exosomes released from acute myeloid leukemia cells have a higher load of TGFβ1, which binds to receptors in the recipient cells and helps in tumor progression through ERK, AKT, and anti-apoptotic pathways [14]. The paracrine mechanisms by which exosome dependent intercellular communications have been studied in detail, for example, glioma cells transfer exosomes rich in oncogenic receptor EGFRvIII to glioma cells lacking the receptor and triggers oncogenic signals through the AKT pathway in the recipient cells. A similar mechanism was revealed in breast cancer progression by exosomes harboring PDL-1 [45]. Exosomes derived from tumor cells governs endothelial cell biochemistry and promote excessive angiogenesis in a hypoxic environment. Cancer cell-derived exosomes that contain TSPAN8 and integrin α4 promote over-proliferation and angiogenesis of endothelial cells [46]. Cancer-associated fibroblast exosomes (CAF-Des) with a heavy load of ADAM10 enhance the motility of cancer cells through GTPase-mediated RhoA and NOTCH signaling [47]. The strong impact of exosomes in cancer drug resistance through the high load of miRNAs (miR-30a, miR-222, and miR-100) is well studied in ovarian and melanoma cancer cells [17,21]. Exosomes exert multiple effects on cancer metastasis. Metastasis involves several steps, including cell migration and invasion, circulation via the lymphatic system or blood vessels, intravasation, and extravasation to distant organs’ parenchyma [33]. Exosomes also govern the cancer cell polarity and movement. Fibronectin and integrin-rich exosomes from fibrosarcoma cells facilitate the integrin clustering and modifying of the extracellular matrix components, ultimately promoting cell migration [19]. The exosome-dependent mechanism of cancer metastasis can be easily explained by the seed, soil, and germinate hypothesis. Successful metastasis depends primarily on intrinsic factors of the cancer cells (seed) to initiate genetic changes and target the microenvironment of the target organ (soil) for the survival of the cancer cells in the new organ (germinate).

The engineering of exosomes in cancer theranostic has increased substantially. The functional molecules can be inserted into a cavity or surfaced into the membrane of exosomes for therapeutic use. One of the most promising areas is exosome payloads with lipid-based or conjugated designer drugs for cancer. A small load of hydrophobic molecules with folic acid with sonication and shear force conjugation method helps in the selective target of the lipid layer in the cancer cell [48]. Electroporation is another technique for loading small nucleotide DNA, miRNA, siRNA, and RNAi into the cavity of the exosomes. In this technique, a mild electric field is applied to increase permeability for small molecule drugs and the large biologicals through the exosomal membrane. Hypotonic techniques are also used to incorporate drugs into exosomes with higher loading efficacy but cause pH changes during the dialysis. Therapeutic drugs such as curcumin and doxorubicin can be injected into exosomes by the co-incubation method with advantages of drug availability in the target site without much degradation in the circulation. Shtam and others [34] successfully demonstrated the encapsulation of siRNA in exosomes by using the transfection agent lipofectamine. Other researchers also demonstrated that exosomes loaded with gold nanoparticle (GNP) drugs can selectively kill the lung cancer cells [18,49].

### 2.2. Neurogenerative Diseases and Exosomes

Exosomes and microvesicles are known to have multifactorial roles in the functioning of the central nervous system. The role of exosomes in the transport of the transmissible prion protein (PrP^C^), α-synuclein in Parkinson’s disease and Tau in Aβ Alzheimer’s disease (AD), Huntington’s disease (HD), multiple sclerosis, and traumatic brain injury is well documented [36,50]. Oligomeric forms of Aβ are neurotoxic and found to be closely associated with exosomes. The primary release of exosomes is regulated by the process of depolarization in the cortical neurons and astrocytes. Some neurons such as oligodendrocytes also release exosomes, which extend the beneficial effect towards neurons against neurons stress phenomenon by transporting SOD1, catalase, and synapsin-1 [31]. The genetic and lipid transporters of exosomes also provide a suitable strategy for neurologic disease-based biomarkers as they can be detected in the cerebral spinal fluids. Researchers have identified exosome-related miRNA panels that show expression differences between control and diseased individuals [51]. Autophagic lysosomal dysfunction of neurons in AD is due to the leakage of cathepsins, B and D, and lysosomes associated membrane proteins (LAMP-1), resulting in lysosomal exocytosis. The role of exosomes in synaptic regulation through control of the neurotransmitters and myelin membrane functions is also well documented [52]. The neurons and glial cells orchestrate the central nervous system hemostasis with the help of exosome-mediated neuroimmune communication. The communication is bidirectional mode; neurons sense the inflammatory signals, glial cells counter-sense the signal and prevent the neuropathic changes by a contact-dependent mechanism mediated by exosomes. The player molecules include the neuron-specific transmembrane ligand proteins like, cluster differentiation 200 (CD200) and fractalkine (CX3CL1), and their microglia-specific receptors CD200 receptor (CD200R) and fractalkine receptor (CX3CR1) [22]. Exosomes play governing roles in clearing these red spot molecules in brain inflammation conditions.

It is now a point of concern and debate as to how to reconcile beneficial and harmful aspects of exosomes in brain resilience during the central nervous system inflammation condition by exosome bioengineering aspects. Secretion of exosomes is an additional benefit of unburdening the neuron’s lysosomal damage system by delivering endosomal-lysosomal material into the extracellular space, where other cell types may contribute to the degradation of neuronal debris. One possibility is that maintaining robust neuronal exosome production may prevent or mitigate endosomal and lysosomal abnormalities linked to aging and neurodegenerative diseases. Another target of controlling brain disease is hemostasis control of reactive oxygen stress in neurons. Antioxidant-decorated miRNA encapsulated exosomes can regulate neurodegenerative diseases. However, there is insufficient research data to uncover the precise mechanisms of exosome engineering in CNS pathology. Detailed guidelines on clinical limitations in the context of exosomes ae available in the recently published guideline “Minimal information for studies of extracellular vesicles 2018” [MISEV2018] [15].

### 2.3. Cardiovascular Complications and Exosomes

Cardiovascular homeostasis is tightly controlled by a complex network and interactions of various cell types, such as cardiomyocytes, cardiac fibroblasts, endothelial cells, macrophages, neurons, and several immune cells. All these cells are vital players in the maladaptive function of the heart characterized by cardiac hypertrophic growth, capillary refractions, myocardial scar formation, interstitial fibrosis, and exacerbated inflammation in myocardial cells [30]. The role of exosomes in the cardiovascular scenario primarily focuses on non-coding RNAs (ncRNAs) [23]. Several types of ncRNAs are studied as per the sizes (small miRNAs and long lncRNAs), shape (linear and circular circRNAs), and position in cell (nucleolar snoRNAs and cytoplasmic). The high population of circRNAs in ischemic myocardium was associated with the metabolic biogenesis of vesicle generation. The depleted circRNAs ignited transforming growth factor-beta (TGF-β) signaling in cardiomyocytes, commonly associated with myocardial fibrosis and inflammatory condition [29]. Although ncRNAs can travel through gap junctions between the cells, exosome-mediated transfer is also reported [53]. Exosome-mediated ncRNAs are emerging as critical players in the cardiac cellular cross-talk, significantly affecting the cardiac microvasculature hemostasis. Sporadic information is available on the significance of lncRNAs, and circRNAs, as new therapeutic exosome targets. To date, miR-122 loaded is the only ncRNA that has reached a phase II clinical trial through engineering aspects.

### 2.4. Skeletal Muscles, Bones, and Exosomes

Around 30–40% of the body comprises skeletal muscle. High contractile forces disintegrate the sarcolemma during eccentric muscle contraction. Regeneration of the damaged tissue is regulated by types of protein secreation, inflammatory cytokines, miRNAs, and membrane lipids [54]. Exosomes in the bone micro-environment facilitate intercellular communication by targeting the same cell, nearby cells, and distant cells through circulation traffic. Exosomes packed with physiologically active molecules can be used for molecular therapy in musculoskeletal disorders, such as osteoporosis, imperfect osteogenesis, and fracture healing. Several techniques are being developed, including (1) low-velocity spin (300–500 RCF) to remove cells and apoptotic debris, (2) a higher speed spin (1000–20,000 RCF) to eliminate larger vesicles, and finally, (3) high-speed centrifugation (100,000–150,000 RCF) to isolate exosomes from bone-derived fluids. Bone is a major hard tissue with a high remodeling feature regulated by a highly coordinated activity of osteoblasts and osteoclasts. Osteoblast-derived exosomes communicate with the osteoclasts with an exchange of growth factors, mRNAs, and miRNAs. Exosomes derived from mineralized osteoblasts contain several miRNAs, such as miR-1192, miR-680, miR-302, that are delivered to bone marrow stromal cells (BMRC) to initiate osteogenic differentiation [24]. MiRNAs such as miR-185 transported through exosomes contribute to osteoporosis and age-dependent bone pathology, especially in older women [55]. It is now clear that inter-organ communication is largely managed by circulating micro-vesicles where miRNAs play a vital role. MiR-218 is a crucial molecule in the signal-amplification circuit-dependent mechanism during osteogenic differentiation in human adipose tissue-derived stem cell differentiation [26]. The role of ncRNAs as therapeutics for musculoskeletal diseases, including osteoarthritis, rheumatoid arthritis, and muscular dystrophies have been identified [25,56]. However, the precise mechanisms of delivering the ncRNAs to the target tissues remain to be investigated.

Currently, there is no long-term effective treatment available for osteoarthritis. Clinical experts prescribe pain killers, stiffness reducers, and surgical joint replacement as options to manage joint dysfunction. Delivering antagonists to marker miRNAs, or targeting the factors upstream of these miRNAs that trigger their expression (e.g., reactive oxygen species, reactive nitrogen species), may represent innovative approaches for slowing the loss of bone volume with age progression, especially during the hip joint pathogenesis. In bone regeneration therapy, cell-free alternates are being attempted with exosomes. Exosomes loaded with growth factors, bioactive molecules, cytokines, and chemokines can be considered novel alternatives to cell-free therapy in bone [57]. Exosomes loaded with anti-inflammatory agents that inhibit the over-activation of macrophages or nano-glucocorticoid agents may serve as an alternate strategy to combat bone resorption disorders [58]. Inhibiting the nucleotide-binding domain and leucine-rich repeat-containing family, pyrin domain-containing 3 (NLRP3) inflammasome through the nano-engineering principle can also be an alternate tool in bone remodeling [59].

### 2.5. Inflammatory Disorders and Exosomes

Tissue inflammation is defined as the caveat response of cells to harmful stimuli, such as exposure to pathogens, injuries, or environmental stress. Immediately upon stimulus, cellular hemostasis ignites the defense mechanism to clear the intruders and dead cells and initiate the tissue repair through communication between resident cells and immune cells. PRRs and TLRs first sense the bacteria, viruses, parasites, and fungi, and initiate the synthesis and release of various inflammatory cytokines and chemokines. There are several subtypes of TLRs with diverse functions. TLRs 1, 2, 4, 5, and 6 are expressed on the cell surface, while TLRs 3, 7, 8, and 9 are found in intracellular compartments with recognition patterns for different microbial components, such as lipoproteins/lipopeptides, peptidoglycan, glycosylphosphatidylinositol, phenol-soluble modulin, zymosan, and glycolipids [60]. Exosomes act as carriers of the inflammatory signaling molecules to macrophages [61,62] and play important roles in mediating and resolving inflammation. The guard cells, including macrophages and mast cells, ignite the inflammatory signaling pathways by releasing chemokines and cytokines, which eventually attract the neutrophil extravasation and subsequent clearance of pathogens [15,27]. Dendrite cells, neutrophils, T-cells, and macrophages can secrete and receive the inflammatory mediator molecules. The trans- communication between donor and recipient cells dictates the degree of inflammation directly proportional to the exosomal load. Scientific databases such as ExoCarta (Exocarta.org), EVpedia (evpedia.info), Vesiclepedia (microvesicles.org) and miRandola (mirandola.iit.cnr.it) are loaded with the latest updates on exosomes and inflammation [63,64,65,66]. The role of exosomes in inflammatory-related cancer pathogenesis, neurogenerative disease, musculoskeletal disorder, cardio-pulmonary etiology, and chronic inflammatory skin diseases is widely reported [67,68,69]. An imbalance between pro and anti-inflammatory mediators also triggers inflammatory bowel diseases by carrying the Ras related proteins, such as 27A (RAB27A) and RAB27B [70]. In the scenario of respiratory tract diseases, such as asthma and allergic sensitization, exosomes perform crucial pathophysiological functions in developing and continuing the pathogenic mechanisms in the alveolar cells, goblet cells, and airway epithelial cells [15,71]. The airway epithelium is the first defense barrier against external irritants and toxins, including air pollutants, smoke, allergens, and pathogens. The surface layer of bronchi and alveoli in airway epithelial cells (AECs) are in direct contact with the external environment. Hence, these cells immediately contribute to the establishment and progression of asthma and allergic airway inflammation and activate the over-secretion of exosomes. The miRNA Let-7, a key marker of asthma, is carried by exosomes to neighboring cells. Direct binding of Let-7 to cytokine IL-13 at the 3′-UTR region has been observed in airway inflammation [37]. However, different unknown signaling biochemical pathways about the specific role of exosome hypersecretion in airway inflammation still need to be defined. Exosomes are “professional transporters and carriers of molecules” to the target cells, thus making them a possible target for the therapeutic delivery of small interference RNAs (siRNAs), miRNAs, and short hairpin RNAs (shRNAs) during allergic inflammation. miRNA-exosome “omics research” is required to establish the signature database in inflammation research that provides investigators and clinicians with better diagnostic, prophylactic, and therapeutic approaches.

## 3. Empowering of Exosome by Engineering

**Figure 2:**
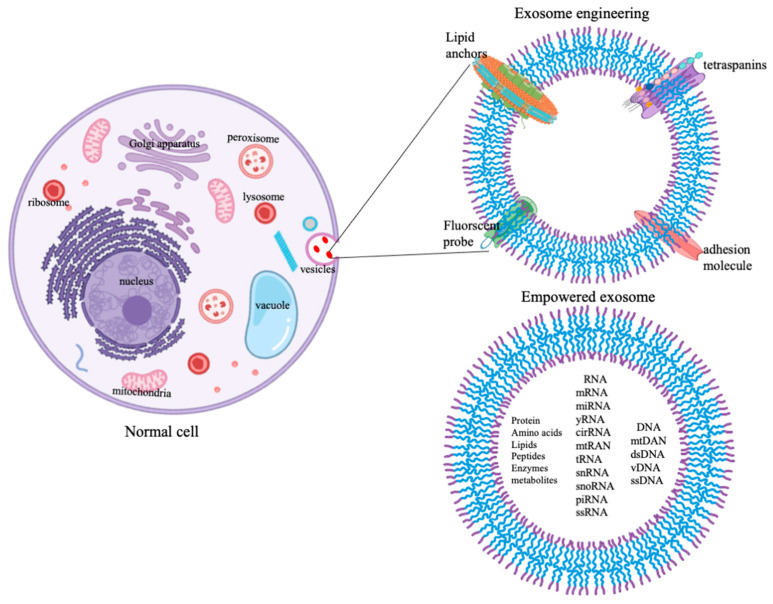
Schematic of exosome engineering.

The strategy for exosome empowerment is depicted in Figure 2. Different cells secrete different exosomes into the circulatory medium in the body. Exosomes have shown great potential as biomarkers for disease diagnostics as well as drug delivery vehicles for therapeutics. The surface protein and lipid composition of exosomes are crucial in redesigning the payload into the cavity of exosomes. Therefore, it is important to study the surface composition of exosomes before evolving the strategy of loading them with drugs. Attempts are made to use the tumor-derived exosomes for transport of chemotherapeutics agents and vaccines for cancer immunotherapy. However, default-loaded molecules, including cathepsin D, adhesion molecules such as vimentin, galectin 3-binding protein, annexin A1, and plasminogen activators, have been associated with tumor-promoting aspects in recipient cells [20]. Furthermore, tumor-derived exosomes exhibit induction of pro-inflammatory cytokines by imparting the monocyte differentiation, induction of myeloid suppressive T cells, and suppressing the lymphoid activation signaling cascade in the tumor microenvironment [72]. These constraints are being abolished by adopting the alternate route of delivering the exosomes derived from natural sources. In this regard, exosomes derived from milk may find a unique place in treating various degenerative diseases, including cancer. Exosome derived from vertebrate milk is a source of countless research. Research on the effect of exosomes isolated from milk derived from various species such as human, bovine, horse, sheep, and goat, in controlling and governing diseases are reported [73]. Milk exosomes can be loaded with hydrophobic and hydrophilic drugs either through simple co-incubation or active incorporation methods, including electroporation, sonication, freeze-thaw cycling, extrusion, and changes in the physicochemical environment (pH and temperature) of the reaction milieu. However, the majority of the studies on drug loading into exosomes are confined to the simple method of incubation. Intended therapeutic benefits of milk exosome delivery in clinical translation require a standardized mode of administration of exosomes.

Another route of exosome engineering is surface engineering with antibodies, ligands, aptamers, and other macromolecules that are specific to the specific target cells. Simple lipo-binding techniques are being discussed for drug load enhancement at the surface [32,74]. Recently, the click-chemistry technique involving the conjugation of 1-Ethyl-3-(3-dimethyl aminopropyl) carbodiimide -N-hydroxy succinimide and azido group was standardized to modify the surface chemistry of the exosomes. Electrostatic interaction, i.e., the fusion of positively charged molecules such as pullulan, lipids, and polymers with negatively charged exosomal membrane proteins, is also being tested. Surface coronation with self-assembling transferrin, folic acid, and hyaluronic acid is also being tried [7,28,43,56]. In cancer biology, exosomes comprising both chemotherapeutic drugs and contrast materials (imaging/diagnostic agents) are gaining importance as theranostic agents. Loading of paramagnetic FeO_2_ nanoparticles, defined as fexosomes in cancer imaging (MRI techniques), and gold nanosomes for CT/PET imaging require further insight for the development of “exo-theranostic” composite. Liver injury and clearance of unwanted metabolites from liver cells is a challenging issue in hepato-pathology. The normal liver contains different cells, such as hepatocytes, hepatic stellate cells (HSCs), and Kupffer cells. Liver fibrosis occurs as a direct consequence of the activation of the HSCs with the overproduction of cytokines and growth factors. HSCs derived exosomes carry marker molecules, such as carboxylesterase-1 (CES1), alcohol dehydrogenase-1 (ADH1), glutathione S-transferase, apolipoprotein A-1 (APOA1), albumin (ALB), haptoglobin (HP), and miRNA-122 [75]. Various cytokines (TGF-β, PDGF, IL-1β, IL-6, IL-13, IL-33, and TNF-α) play essential roles in liver fibrosis. Factors such as alcohol consumption, viral infections, metabolic disorders, toxins, obesity, steatosis, and cholestasis are also involved in liver fibrosis. The delivery of a nano-based drugs formulated using liposomes and polymers is gaining attention as a pharmacotherapy strategy to control liver diseases. Tailoring the nanomedicine with exosomes to target the liver cells and delivering the potent specific pharmaco-immune modulator has a bright future.

An interesting approach could be dissecting the exosome membrane protein, tetraspanins. This superfamily protein governs and organizes the membrane architecture by a mechanism termed ‘tetraspanins enriched microdomains’ by cluster formation and interacting with transmembrane cytosolic signaling proteins [35]. Among tetraspanins, CD9, CD63, CD81, CD82, and CD151, are present in several tissues, while Tssc6, CD37, and Cd53 are specific to hematopoietic cells [15,76]. Aptamers, also known as chemical antibodies with single-stranded DNA or RNA with 3D globe-like structure, are synthesized by PCR-based cell SELEX (systematic evolution of ligand by exponential enrichment) method [77]. The aptasensor technology is highly sensitive with rapid response and hence is beneficial as a nanoprobe in exosome aided early detection of cancers. Exosomes labeled with aptamers can be used to identify the diseased cell. Nano-capturing platforms using exosomes with aptamers specific for target proteins can help as aptasensor diagnostic tools.

## 4. Summary and Future Road Map

Following a humble beginning two decades ago, exosome engineering is now paving the way for developing new and effective tools in disease diagnostics and therapeutics. The details of the schematic of drug loading engineering are tabulated in Table 2.

Chemical and nano-engineering efforts have led to designing designer exosomes with an improved pharmacokinetic profile of specific diseases. Starting from the systematic evolution of ligands by exponential enrichment (SELEX) technique, research is now focused on developing the nPLEX technology with higher sensitivity for disease detection, fluorescent-based micro-fluid chips for early identification, and interferometric imaging hybrids for imaging techniques using next generation exosomes. More research is required on the aspects of exosome quantification and in vivo imaging techniques. Gateway research by Gupta and others [81] forecasts a new avenue of live imaging using luciferase conjugated tetraspanins termed as nanoLuc or ThermoLuc method for live distribution of exosomes in tissue levels. Non-invasive tracking of exosomes through the luciferase system yields higher results due to the high pitch and intensity and half-life of luciferase systems at the sub-tissue level. A step higher strategy such as double conjugation of tetraspanins rich exosome with luciferase and lipophilic fluorescent dyes could yield better results. Another avenue is acousto-fluidics; combining acoustics material in biocompatible fluids with nano-drug-loaded exosomes may serve as promising tools in bio-medical sensor detection in protein separation [82]. The functional efficacy of exosome-based delivery of drugs depends on the nature of the producer cells. Optimizing the safety, efficacy, and cost-effectiveness of exosome-guided therapy requires the source of exosome-producing cells and cell stimulation. For example, a unique material that stimulates the membrane proteins and overproduction of exosomes in the donor cell without countering the safety of the engineered exosomes is a benchmark demand of exosome-mediated drug delivery. However, key challenges persist, particularly in exosome heterogeneity in terms of exosome loading patterns. Therefore, the next decade will evidence more tremendous efforts, particularly on developing the membrane modification techniques in exosomes and smartly controlling the loading patterns.

## Figures and Tables

**Table 1:** Functions of exosomes.

Cell Type/Tissue/Host Nature	Function	Reference
Breast cancer-derived	Metastasis protein transport	[14]
Virology	COVID-19 infection	[11]
Host-pathogen interaction	Toxic transport	[13,15]
Fibroblast to cancer cell	Communication	[16]
Serum	miRNA transport	[17]
Normal cell exosomes	Nanoparticle delivery	[18]
Metastasis breast cancer	GTPase and Rab27B delivery	[19,20]
Ovarian cancer cell	Lysosomal delivery	[21]
Microglia	Neuron signal communication	[22]
Cardiomyocytes	Non-coding RNA signals	[23]
Osteoblast cells	MiR-31 transport	[24,25,26]
Neutrophils	Inflammatory signals	[27,28]
Fibroblast	Deviation in TGF beta signaling	[29]
Cardiac tissue	Heart failure	[30]
Neuronal cells	Neuron diseases	[22,31]
Endothelial cell	Endocytosis	[32]
Melanoma	miRNA circulation	[17]
Alveolar cell	Cancer metastasis	[33]
Normal cell	Exogenous siRNA transport	[34,35]
Neuronal cell	Alzheimer disease	[36]
Oligodendroglia	Cell communication	[31]
Mesenchymal stem cell	miR-31 and miR-28 transport	[24,26]
Myeloid leukemia	Tumor growth factor communication	[14]
Osteoclast	miR-28 loading	[26]
Lung airway cell	Let-7 regulation	[37]
Adipose-derived stem cells	Angiogenesis	[38]

**Table 2:** Metabolites loading patterns during exosome engineering.

Cargo Types	Technique of Loading	Principle	Merits/Demerits
Synthetic drugs	Incubation	Membrane diffusion	Simple and easy [4]
Nucleic acid, peptides,	Transfection	Gene manupulation	Efficiency coefficient to be standardized [78]
Drugs, materials	Polymerisation	Issues of membrane pore formation	Very high loading efficiency [79]
Nucleic acids, RNA, and Peptides	Freeze-Thaw	Membrane fusion with liposomes	Moderate loading [43]
Proteins, Peptides, and Materials	Surfactant	Membrane fusion and nanopore	Very effective and high loading [80]
RNAs and DNAs	Dialysis and hybridization	Issues of rapid pH changes in the medium	Easy and less time consuming [18]
Nanomaterials	Hybridization	Chemo-biological reaction	Stability [56]

## Data Availability

This manuscript does not have data sharing options. Now new data was generated; the manuscript describes theoretical aspects.
